# The Hippo Pathway Effector Transcriptional Co-activator With PDZ-Binding Motif Correlates With Clinical Prognosis and Immune Infiltration in Colorectal Cancer

**DOI:** 10.3389/fmed.2022.888093

**Published:** 2022-07-05

**Authors:** Yutong Wang, Hui Nie, Huiling Li, Zhiming Liao, Xuejie Yang, Xiaoyun He, Jian Ma, Jianhua Zhou, Chunlin Ou

**Affiliations:** ^1^Department of Pathology, Xiangya Hospital, Central South University, Changsha, China; ^2^Department of Pathology, Rizhao City People’s Hospital, Rizhao, China; ^3^Department of Ultrasound Imaging, Xiangya Hospital, Central South University, Changsha, China; ^4^Cancer Research Institute and School of Basic Medical Science, Central South University, Changsha, China; ^5^National Clinical Research Center for Geriatric Disorders, Xiangya Hospital, Central South University, Changsha, China

**Keywords:** colorectal cancer, TAZ, Hippo pathway, tumor immunology, immune infiltration, biomarker

## Abstract

The transcriptional co-activator with PDZ-binding motif (TAZ) is a downstream effector of the Hippo pathway. It has been identified as an oncogene in certain tumor types; however, the function and role of TAZ in colorectal cancer (CRC) has not been illustrated. Here, we aimed to analyze the expression and role of TAZ in CRC. In this study, we investigated the expression level of TAZ in 127 CRC and matched adjacent normal tissues by immunohistochemistry (IHC) and analyzed its correlation with clinicopathological characteristics in CRC. Moreover, we further analyzed the role of TAZ in the CRC-associated immunology using integrative bioinformatic analyses. The cBioPortal and WebGestalt database were used to analyze the co-expressed genes and related pathways of TAZ in CRC by gene ontology (GO) and KEGG enrichment analyses. Meanwhile, the correlations between TAZ and the infiltrating immune cells and gene markers were analyzed by TIMER database. Our study revealed that TAZ expression is higher in CRC tissues than in matched adjacent non-tumor tissues. In addition, CRC patients with higher TAZ expression demonstrated poor overall survival (OS) and recurrent-free survival rates as compared to CRC patients with lower expression of TAZ. Furthermore, the TAZ expression was identified to closely associate with the immune infiltration of CD4 + T, CD8 + T, and B cells. Taken together, our findings suggest that TAZ may serve as a promising prognostic biomarker and therapeutic target in CRC.

## Introduction

Hippo signaling pathway is a highly conserved intracellular communication network in multiple species. It was originally discovered from Hippo kinase in Drosophila and mainly comprises the upstream activated factors, such as FAT4 and DACHS; core kinase molecules, such as MST1/2 and LATS1/2; and co-transcription factors including YAP and transcriptional co-activator with PDZ-binding motif (TAZ) (1, 2). When Hippo signaling is inhibited, TAZ is translocated to the nucleus from the cytoplasm and acts as a co-transcription factor with other transcription factors such as TEADs, RUNXs to form transcription complexes, thereby regulating the transcriptional expression of a series of downstream target genes (e.g., TEADs, Runxs) (3, 4). Recent studies have reported that the activation of TAZ is closely linked with tumorigenesis as it regulates a series of malignant biological behaviors, such as malignant proliferation, invasion, metastasis, and chemotherapy resistance (5–8).

The Hippo effector TAZ is short for “TAZ.” It is encoded by the *WWTR1* gene and located at 3q25 in humans (9). In recent years, TAZ has been identified as an oncogene in several tumor types such as breast, lung, prostate, and ovarian cancers (5–8). Maugeri-Sacca et al. (10) have demonstrated that the high expression of TAZ is associated with the promotion of epithelial-mesenchymal transition (EMT) and chemotherapy resistance in breast cancer. Furthermore, the activation of TAZ is also connected to various cancer hallmarks, such as EMT in thyroid carcinoma (11), glutamine metabolism in breast cancer (12), and chemotherapy resistance in non-small-cell lung cancer (13). Moreover, the expression of TAZ holds prospective prognosis value in certain tumor types. Van Haele et al. (14) have established that the nuclear expression of TAZ is an independent predictor of poor recurrence-free survival (RFS) and overall survival (OS) in hepatocellular carcinoma patients. In colorectal cancer (CRC), several previous studies have reported the association of TAZ with prognosis and tumor aggressiveness (15–17). Moreover, recent studies demonstrated that Hippo pathway was associated with the tumor immunity (18, 19). TAZ may be served as a signal hub that link the cancer cells and immune cells, which can influence the formation of tumor microenvironment (TME), thereby promoting the immune escape (20, 21). However, the specific function and immune role of TAZ in CRC has not been fully elucidated.

Accordingly, in this study, we investigated the expression of TAZ in CRC and matched normal tissues for analyzing the correlation between TAZ expression and clinicopathological characteristics in CRC. Furthermore, we analyzed the role of TAZ in the CRC-associated immunology using an integrative bioinformatic analyses. Thus, the aim of this study was to assess the clinical significance and potential role of TAZ, and to analyze the relationship between TAZ expression and immune infiltration in CRC.

## Materials and Methods

### Tissue Samples

A total of 127 pairs of paraffin-embedded archived CRC specimens and matched adjacent normal tissue samples were obtained from Xiangya Hospital (Changsha, P. R. China). All the CRC tumor specimens obtained after surgical resection were collected between June 2016 and May 2019. None of the patients had received any treatment such as chemotherapy, radiotherapy, or immunotherapy, prior to resection. The clinical CRC specimens were collected with permission from the hospital Research Ethics Board of Xiangya Hospital of Central South University.

### Immunohistochemistry

The immunohistochemistry (IHC) procedures were performed using classical biotin–streptavidin–peroxidase staining protocols (22). TAZ expression was detected using anti-TAZ antibody (diluted 1:50; Catalog no: 23306-1-AP; Proteintech Group, Inc, United States). Five random high-power fields were chosen and more than 500 cells were analyzed per field for assessing TAZ expression. The sample TAZ expression scores were based on both the intensity of staining and the proportion of positively stained tumor tissues, with staining intensity scores being either 0 (negative), 1 (weak), 2 (moderate), or 3 (strong); and staining area scores being either 0 (0%), 1 (1–25%), 2 (26–50%), 3 (51–75%), or 4 (76–100%). The final staining score of each specimen was calculated as the product of staining intensity and the staining area scores. The final staining score of 2 or above was considered as positive TAZ expression whereas a staining score of less than 2 was considered as negative TAZ expression (23). The data were subjected to statistical analyses and the associated results are shown in the Supplementary Table 1.

### Data Collection and Integrative Bioinformatic Analyses

#### Gene Expression Omnibus Database Analysis

Colorectal cancer datasets were deposited in the Gene Expression Omnibus (GEO) database: GSE9348 (24), GSE22598 (25), GSE18105 (26), and GSE39582 (27) (these datasets were generated using the Affymetrix Human Genome U133 Plus 2.0 platform). The GSE9348 dataset has 70 primary CRC samples and 12 normal colon samples, GSE22598 has 17 pairs of CRC and adjacent non-tumor tissues, GSE18105 has 67 primary CRC samples and 44 metastatic CRC samples, and GSE39582 contains clinical follow-up data for 574 CRC samples.

### TIMER Database Analysis

TIMER^1^ is a comprehensive analysis software for investigating the gene expression and immune infiltration in various tumor types (28). TIMER database contains 457 colon adenocarcinoma (COAD) tumor samples and 41 normal samples, and 166 rectum adenocarcinoma (READ) tumor samples and 10 normal samples. We used TIMER to reveal the correlation between TAZ expression in CRC and the level of infiltration by immune cells. In addition, the correlation of TAZ expression with gene markers of CD4^+^ T, CD8^+^ T, B, and other types of T cells in addition to macrophages, neutrophils, and monocytes were further investigated (29). The gene expression levels were expressed as log_2_ RSEM (RNA-Seq by Expectation-Maximization).

#### UALCAN Analysis

UALCAN^2^ is a user-friendly web resource that contains comprehensive multi-omics data pertaining to various cancers that facilitates the identification of tumor subgroup-specific candidate biomarkers and their validation (30). In the present study, we analyzed the expression of TAZ in CRC patients through UALCAN using cohort stratification on the basis of stage and metastasis status of the disease.

#### Co-expression Network

cBioPortal^3^ integrates data from several databases and independent cancer research projects and facilitates visual examination of multi-dimensional cancer genomics data (31). In the present study, we used it to analyze the co-expressed genes of TAZ in CRC. The subsequent protein-protein interaction (PPI) analysis was carried out using Cytoscape (version 3.7.2)^4^ (32).

#### Enrichment Analysis

Metascape^5^ incorporates comprehensive annotated gene lists and analysis resources for experimental biologists that facilitates extensive analyses of datasets across multiple independent and orthogonal experiments (33). In the present study, we performed gene ontology (GO) enrichment and KEGG pathway enrichment to analyze the co-expressed genes of TAZ in CRC, and visualized it using Metascape.

### Statistical Analysis

The differences between two groups were analyzed with the Student’s *t*-test using statistical analysis software package IBM SPSS Statistics 23.0 (International Business Machines Corporation, NY, United States). Chi-Square test was used to analyze the relationship between TAZ expression and the clinicopathological characteristics of CRC patients. The correlation of TAZ expression with immune infiltration and characteristic markers of immune cells were analyzed with Spearman’s Rank correlation. Results were considered statistically significant at *P*-value < 0.05.

## Results

### Transcriptional Co-activator With PDZ-Binding Motif Is Overexpressed in Colorectal Cancer Tissues

**FIGURE 1:**
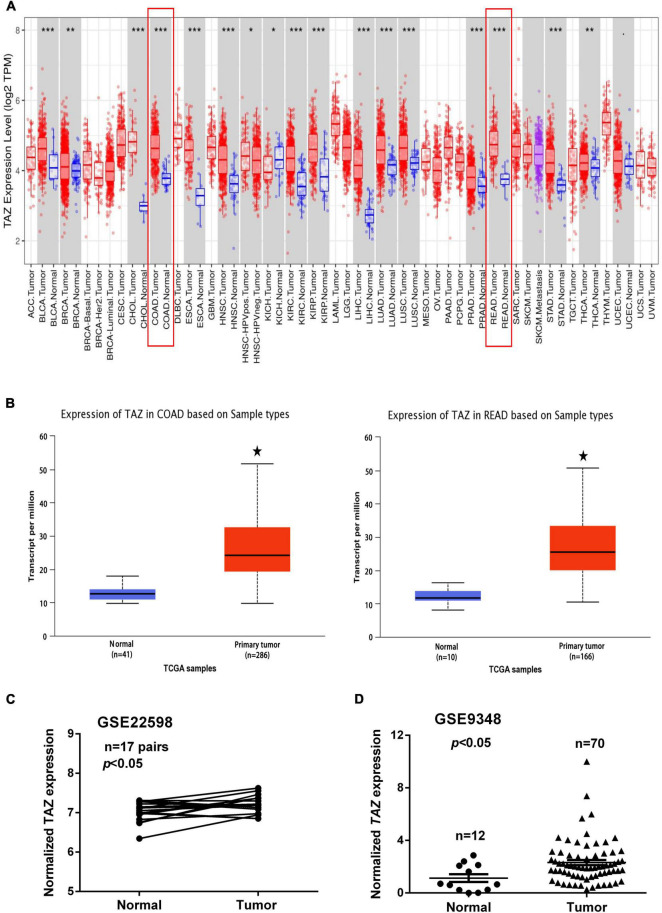
Transcriptional co-activator with PDZ-binding motif (TAZ) is overexpressed in colorectal cancer (CRC). **(A)** Relative expression of TAZ in different cancers as compared with that in adjacent normal tissues as per the analysis using TIMER database. **(B)** Box plot showing expression of TAZ in colon adenocarcinoma (COAD) and rectum adenocarcinoma (READ) patients as compared with that in normal healthy individuals generated using the UALCAN database. **(C)** #GSE22598 (containing 17 pairs of CRC tissues and corresponding normal colorectal tissues) and **(D)** #GSE9348 (containing 12 normal colon samples and 70 primary CRC samples) from the Gene Expression Omnibus (GEO) database were used to analyze the expression of TAZ. *P < 0.05, **P < 0.05, ***P < 0.001 as compared with control.

To analyze the expression levels of TAZ in various human cancers as compared with those in normal tissues, the mRNA expression of TAZ was analyzed using TIMER database. The results showed that TAZ expression was significantly elevated in bladder urothelial carcinoma (BLCA), breast invasive carcinoma, cholangiocarcinoma (CHOL), esophageal carcinoma (ESCA), head and neck cancer (HNSC), kidney renal clear cell carcinoma, kidney renal papillary cell carcinoma (KIRP), liver hepatocellular carcinoma, lung adenocarcinoma, lung squamous cell carcinoma (LUSC), prostate adenocarcinoma, stomach adenocarcinoma, thyroid carcinoma, and especially in COAD and READ (Figure 1A and Supplementary Figure 1A).

**FIGURE 2:**
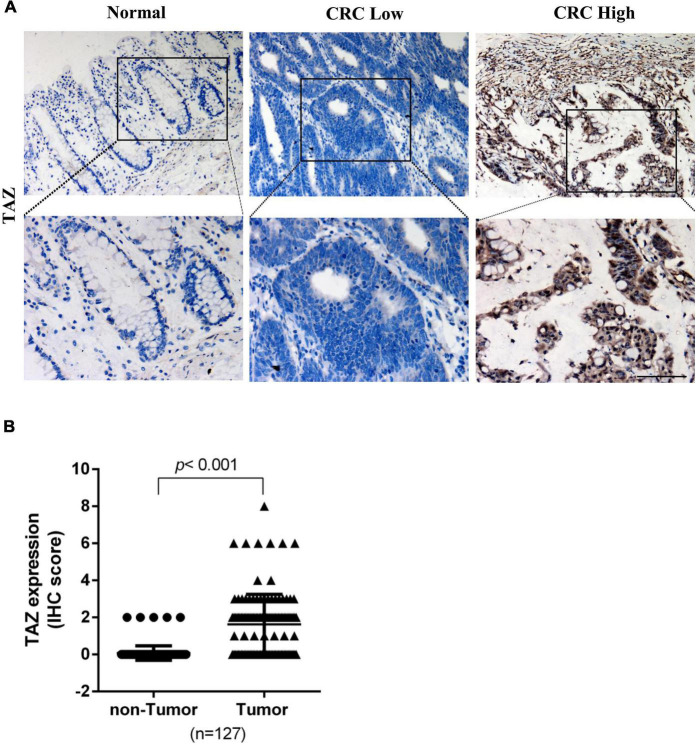
Transcriptional co-activator with PDZ-binding motif (TAZ) expression is up-regulated in colorectal cancer (CRC) tissues. **(A)** Immunohistochemistry (IHC) staining showing the expression of TAZ in matched normal adjacent or CRC tumor tissues (200 × magnification). **(B)** Expression scores of TAZ proteins expression in 127 pairs of CRC tumor tissues and corresponding adjacent normal mucosa tissues.

To further determine the expression of TAZ in CRC, we first employed the UALCAN database to analyze the expression of TAZ in COAD and READ (Figure 1B, *P* < 0.05). Moreover, we analyzed two online GEO datasets (#GSE22598 and GSE9348) based on the Affymetrix Human Genome U133 Plus 2.0 platform. TAZ was significantly upregulated in CRC tissues compared with non-tumor tissues (Figures 1C,D, *P* < 0.05). Subsequently, IHC was performed to validate the protein expression of TAZ in 127 pairs of CRC tumor tissues and corresponding adjacent normal mucosa tissues. IHC staining analysis revealed that TAZ was mainly localized in the nucleus of cancer cells in the tissues analyzed, as indicated by brown staining (Figure 2A). After the statistical analysis, TAZ was also expressed significantly highly in CRC tissues than in the adjacent non-tumor tissues as revealed by the Student’s *t*-test (Figure 2B, *P* < 0.001).

### Transcriptional Co-activator With PDZ-Binding Motif Expression With Clinical Characteristics and Prognosis in Colorectal Cancer

**FIGURE 3:**
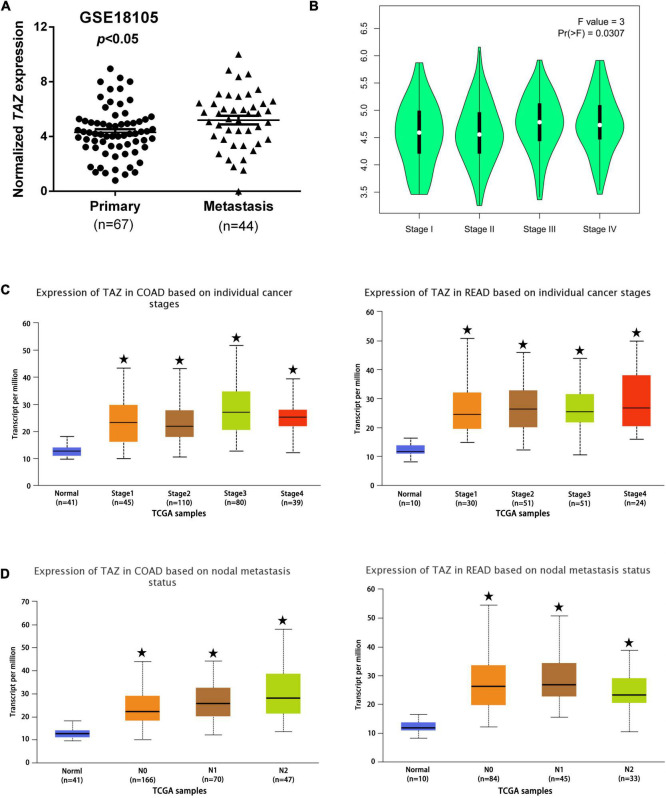
Correlation of transcriptional co-activator with PDZ-binding motif (TAZ) expression with clinicopathological features of CRC patients. **(A)** #GSE18105 [containing 67 primary colorectal cancer (CRC) samples and 44 metastatic CRC samples] from the Gene Expression Omnibus (GEO) database were used to analyze the expression of TAZ in primary and metastatic CRC tissues. **(B)** The Gene Expression Profiling Interactive Analysis (GEPIA) database was used to analyze the association between TAZ expression and the pathological stages of CRC. **(C,D)** UALCAN database was used to analyze the association between TAZ expression and the tumor stages and nodal metastasis status of CRC. *P < 0.05 as compared with control.

To analyze the correlation between TAZ expression and clinicopathological features of CRC, we assessed the correlation between TAZ expression and distant metastasis in CRC tissues by one GEO dataset (#GSE18105). The analysis demonstrated that the expression of TAZ was higher in metastatic CRC tissues than in primary CRC tissues (Figure 3A, *P* < 0.05). Furthermore, the relevant gene expression and corresponding patient data of multiple cancer types from TCGA database and were stratified on the basis of individual tumor stage and nodal metastasis. GEPIA database (34) analysis found that the higher expression of TAZ was significantly associated with the pathological stages of CRC (Figure 3B, *P* < 0.05). UALCAN database analysis was performed, which further revealed that TAZ was more highly expressed in the differential tumor stages and nodal metastasis status groups of COAD or READ than in the normal group (Figures 3C,D and Supplementary Figure 1B, *P* < 0.05).

Depending on the staining score of TAZ in the IHC studies, all CRC patients were divided into low and high TAZ expression groups. The integration of corresponding clinical characteristics along with the expression of TAZ revealed their strong correlation with the TAZ high- and TAZ low-expression groups. Statistical analyses revealed that high TAZ expression was significantly associated with the N stage and the differentiation degree of CRC patients (*P* < 0.05, Table 1 and Supplementary Figure 2). These results indicated that TAZ may be a biomarker for CRC. Furthermore, CRC was divided into four subgroups by the consensus molecular subtypes (CMS) classifications, named as CMS1, CMS2, CMS3, and CMS4 (35). Zhang et al. found that TAZ overexpression is related to specific CRC subgroups, such as the CMS4, which was associated with the poor prognosis of CRC patients (36). Therefore, we further analyzed the correlation between TAZ expression and the status of MSI, BRAF, and KRAS in CRC patients. However, the results showed no significant correlations between them (Supplementary Table 2).

**TABLE 1:** Association between the expression of transcriptional co-activator with PDZ-binding motif (TAZ) and colorectal cancer (CRC) patients’ clinicopathological parameters.

Characteristics	N	TAZ		High (%)	χ2	*P*
					
		Low	High			
Gender					0.421	0.571
Male	69	27	42	60.86%		
Female	58	26	32	55.17%		
Age (year)					0.006	0.939
≤60	70	29	41	58.57%		
>60	57	24	33	57.89%		
T stage					1.007	0.879
T1	2	1	1	50.00%		
T2	13	4	9	69.23%		
T3	93	40	53	56.99%		
T4	19	8	11	57.90%		
N stage					4.115	0.043*
N0	68	34	34	50.00%		
N1 + N2	59	19	40	67.7%		
Peripheral nerve infiltration					0.078	0.780
No	104	44	60	57.69%		
Yes	23	9	14	60.86%		
Venous invasion					0.117	0.732
No	86	35	51	59.30%		
Yes	41	18	23	56.09%		
Tumor size (cm)					1.254	0.263
≤5	84	38	46	55.95%		
>5	43	15	28	65.12%		
Differentiation degree					8.477	0.013*
Poorly	21	3	18	85.71%		
Moderately	98	47	51	52.04%		
Well	8	3	5	62.50%		

*P < 0.05.

**FIGURE 4:**
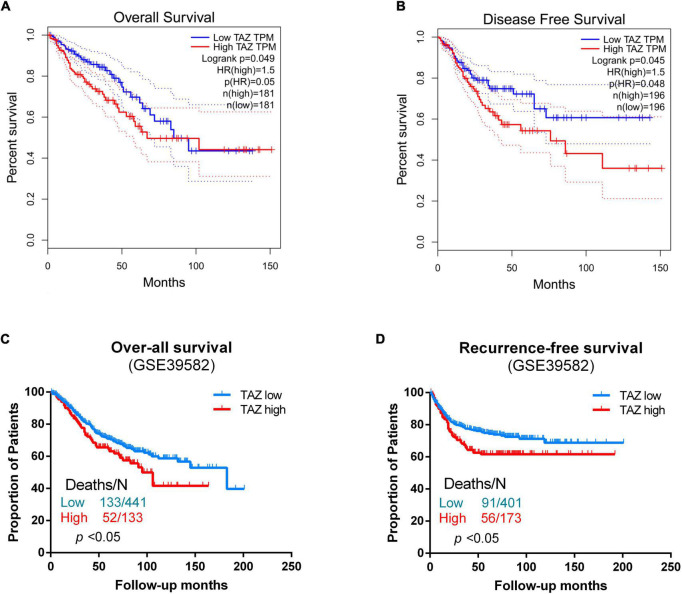
Correlation of transcriptional co-activator with PDZ-binding motif (TAZ) expression with survival status of colorectal cancer (CRC) patients. **(A,B)** GEPIA database was used to analyze the clinical impact of TAZ expression patterns on the overall survival (OS) and disease-free survival (DFS) of CRC patients. **(C)** Kaplan-Meier analysis showing OS curves for CRC patients with different expression levels of TAZ; statistical significance was assessed by log-rank tests (#GSE39582, the specimen was divided into two groups: group 1, low TAZ expression, *n* = 441; group 2, high TAZ expression, *n* = 133). **(D)** Kaplan-Meier analysis showing recurrence-free survival (RFS) curves for CRC patients with different expression levels of TAZ; statistical significance was assessed by log-rank tests (#GSE39582, the specimen was divided into two groups: group 1, low TAZ expression, *n* = 401; group 2, high TAZ expression, *n* = 173).

By analyzing the public CRC samples in the GEPIA database, we found that the high TAZ expression was associated with poor OS and disease-free survival (DFS) (Figures 4A,B, *P* < 0.05). Furthermore, we also examined the association between TAZ expression levels and OS and RFS in the GSE39582 database using Kaplan-Meier analysis with log-rank tests. The results revealed that patients with high TAZ expression levels had lower OS and RFS (Figures 4C,D, *P* < 0.05). Collectively, these data indicate that TAZ can serve as a potential prognostic marker for CRC patients.

### Transcriptional Co-activator With PDZ-Binding Motif Co-expression Network and Its Functional Enrichment Analysis in Colorectal Cancer

**FIGURE 5:**
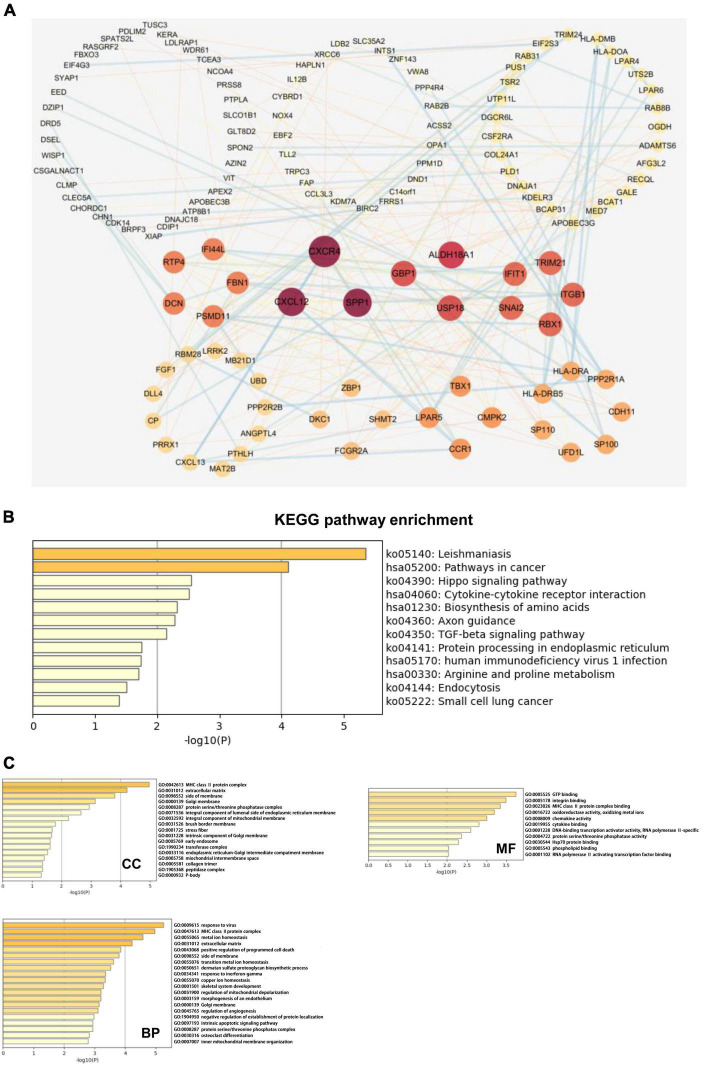
Transcriptional co-activator with PDZ-binding motif (TAZ) co-expression network and pathway enrichment analysis. **(A)** The protein-protein interaction (PPI) network of TAZ expression which is analyzed with cBioPortal database and Cytoscape software. **(B)** The analysis of KEGG enrichment pathway using Metascape database. **(C)** The analysis of gene ontology (GO) cell components (CC), molecular functions (MF) and biological processes (BP) using Metascape database.

We next explored the biological functions of TAZ using the cBioPortal and Cytoscape database. Firstly, we downloaded co-expressed genes of TAZ in CRC from the cBioPortal database, and then screened them based on the absolute value of | log2FC| > 1.2 and *P* < 0.05. Finally, a total of 254 aberrantly expressed genes were obtained (Supplementary Table 3). We next employed STRING and Cytoscape database to illustrate the protein-protein interaction network (PPI network). The results of this analysis revealed the genes, including C-X-C chemokine ligand 12 (*CXCL12*), C-X-C chemokine receptor 4 (*CXCR4*), and secreted phosphoprotein 1 (*SPP1*), that were most closely co-expressed with TAZ (Figure 5A). Further, GO and KEGG pathway analyses were performed using Metascape. The results of KEGG pathway enrichment analysis demonstrated that the activated pathways correlated with TAZ expression included “Leishmaniasis,” “Pathways in cancer,” “Hippo signaling pathway,” and “Cytokine-cytokine receptor interaction.” In addition, the co-expressed genes also demonstrated a certain level of enrichment in “TGF-beta signaling pathway” and “human immunodeficiency virus 1 infection,” which indicated that TAZ might be involved in immunological processes associated with CRC (Figure 5B). Meanwhile, the results of GO analysis revealed that the co-expression genes of TAZ were also enriched in the “MHC class II protein complex,” “response to virus,” and “GTP binding” pathways (Figure 5C), which have been reported to be important in the regulation of tumor-associated immunological processes (37, 38). Taken together, it is reasonable to speculate that TAZ expression is closely related to tumor immune-associated molecules and signaling pathways in CRC tissues.

### Correlation of Transcriptional Co-activator With PDZ-Binding Motif Expression With Immune Infiltration in Colorectal Cancer

**FIGURE 6:**
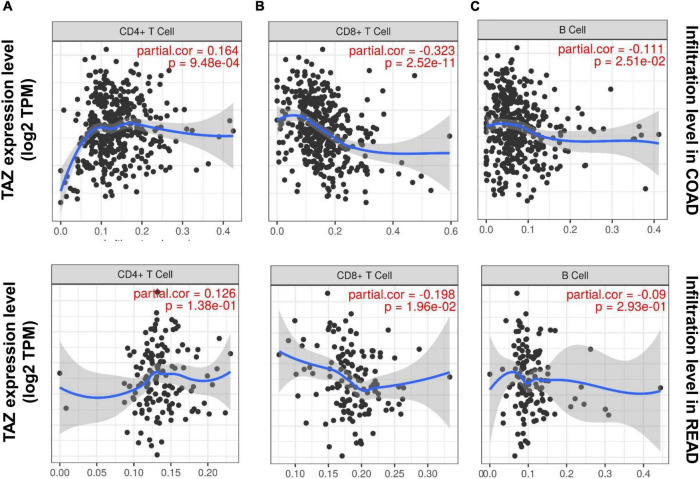
Correlation of transcriptional co-activator with PDZ-binding motif (TAZ) expression with immune infiltration in colorectal cancer (CRC). The correlation between the expression of TAZ and the immune infiltration level of CD4^+^ T cells **(A)**, CD8^+^ T cells **(B)**, and B cells **(C)** in colon adenocarcinoma (COAD) and rectum adenocarcinoma (READ) by the TIMER database.

Tumor-infiltrating cells play critical roles in tumor metastasis, with multiple reports indicating immune infiltration to be an independent factor associated with CRC prognosis, as well as an indication for the treatment interventions employing CRC immunotherapy (39). In the KEGG enrichment analysis, we found that the co-expression genes of TAZ were associated with the immune process. Consequently, we further explored the relationship between tumor infiltration and TAZ expression using TIMER database. Our results showed that the expression level of TAZ was significantly correlated with the infiltration level of CD4^+^ T cells (*r* = 0.164, *P* < 0.05), CD8^+^ T cells (*r* = −0.323, *P* < 0.05), and B cells (*r* = −0.111, *P* < 0.05) in COAD, in addition to the infiltration level of CD8^+^ T cells (*r* = −0.198, *P* < 0.05) in READ (Figure 6). Thus, TAZ expression was significantly correlated with immune cell infiltration in CRC.

**FIGURE 7:**
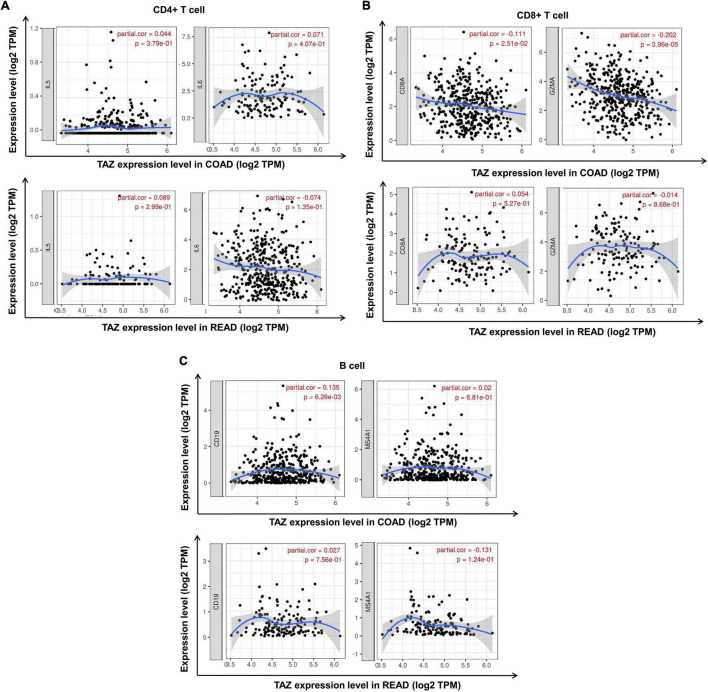
Correlation between the expression of transcriptional co-activator with PDZ-binding motif (TAZ) expression and tumor immune cell markers in colorectal cancer (CRC) by the TIMER database. **(A)** Correlation between the expression of TAZ and CD4^+^ T cell markers, IL5 and IL6 in colon adenocarcinoma (COAD) and rectum adenocarcinoma (READ), respectively. **(B)** Correlation between the expression of TAZ and CD8^+^ T cell markers, CD8A and GZMA in COAD and READ, respectively. **(C)** Correlation between the expression of TAZ and B cell markers, CD19 and MS4A1, in COAD and READ, respectively.

**FIGURE 8:**
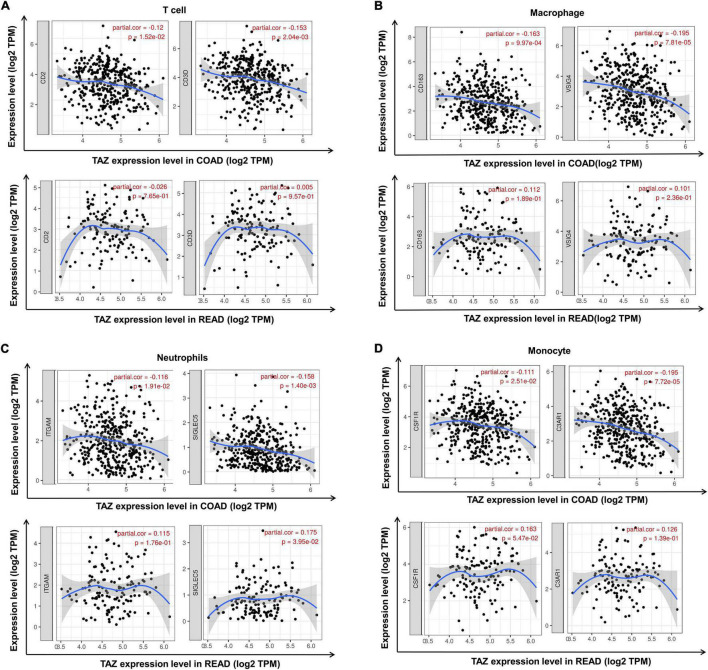
Correlation between the expression of transcriptional co-activator with PDZ-binding motif (TAZ) and additional tumor immune cell markers in colorectal cancer (CRC) by the TIMER database. **(A)** Correlation between the expression of TAZ and T cell markers, CD2 and CD3D in colon adenocarcinoma (COAD) and rectum adenocarcinoma (READ), respectively. **(B)** Correlation between the expression of TAZ and macrophage markers, CD163 and VSIG4 in COAD and READ, respectively. **(C)** Correlation between the expression of TAZ and neutrophil markers, ITGAM and SIGLEC5 in COAD and READ, respectively. **(D)** Correlation between the expression of TAZ and monocyte markers, CSF1R and C3AR1 in COAD and READ, respectively.

Subsequent assessment of the expression of typical immune cell markers in COAD and READ datasets was performed to determine the relationship between TAZ expression and tumor-associated immune processes (Supplementary Table 4). We found that the gene markers of CD8^+^ T cell, B cell, T cell, macrophage, neutrophil and monocyte were comparatively high with TAZ expression in COAD, while only the gene markers of neutrophil and monocyte had significant correlation with TAZ expression level in READ (*P* < 0.05, Figures 7, 8). Taken together, these results indicated the participation of TAZ in potential mechanism in CRC immune regulation.

## Discussion

Colorectal cancer is the third most commonly diagnosed malignant tumor in the world (40, 41), exhibiting high risk of recurrence and distant metastasis. Despite the ever-advancing novel strategies for CRC prevention and treatment (42), the 5-year relative survival rate of postmetastatic CRC is less than 15% (43, 44). Therefore, the pathological mechanisms that promote the tumorigenesis of CRC must be investigated to provide potential targets for effective clinical diagnosis and treatment. As a key downstream effector of Hippo pathway, TAZ has been recently identified as an oncogene in certain tumor types. Moreover, the aberrant expression of TAZ has been identified to be associated with the development of breast, liver, and thyroid cancers (10, 11, 14). Wang et al. (45) have studied the combined amplification of TAZ gene in 33 cancer cell types and found that TAZ exhibits the most significant amplification rate in five common types of squamous cell carcinomas including cervical squamous cell carcinoma (CESC), LUSC, ESCA, HNSC, and BLCA. Furthermore, this study demonstrated that the frequent amplification of YAP1 and TAZ occurred in a mutually exclusive manner in HNSC and CESC, in addition to delineating the molecular mechanism of TAZ being involved in the regulation of squamous cell carcinoma progression. However, the roles and the mechanisms involving TAZ in CRC progression have rarely been investigated. Accordingly, in this study, we performed the integrative bioinformatic analyses of the expression level of TAZ in CRC employing the publicly available cancer gene expression databases. Our results revealed that the mRNA and protein expressions of TAZ were significantly overexpressed in CRC compared to those in the matched normal adjacent tissues. Furthermore, our analysis showed that high TAZ expression was significantly associated with the N stage and degree of differentiation in CRC. Likewise, CRC patients with high TAZ expression levels were also identified to exhibit lower OS and RFS than those with low TAZ expression. Moreover, we found that patients with high TAZ expression levels had lower OS and RFS by the Kaplan-Meier analysis. However, Wang et al. (16) reported that patients with high YAP expression levels had poor prognosis in CRC, especially when TAZ was co-expressed, but at multivariate analysis TAZ alone showed no statistical correlation to prognosis. Because the small sample size limits, this study was not entirely concluded that YAP is a better biomarker or the YAP + TAZ is the best biomarker. Furthermore, Mouillet-Richard et al. (35) found that TAZ mRNA is clearly enriched in CMS4 tumors by the examination of the levels of TAZ by CMS classification, those tumors known to express EMT, TGF-b, and have a worse survival; while, TAZ has a low expression in CMS2 and CMS3 type of tumors. Interestingly, immune infiltration is related to a better outcome in CRC as examined by “immunoscore,” and CMS4 microenvironment is rich in “immune suppressors” (46). In a word, these results indicated the potential of TAZ to become a biomarker in CRC.

The gene co-expression analysis is a high throughput correlation analysis which distinctly illustrates the molecular interaction and systematically corresponds the targeted gene functions with specific biological process. Currently, the gene co-expression network has become a crucial method to search for novel gene markers for CRC targeted therapy (47, 48). In this study, we used the cBioPortal database to analyze the co-expression network of TAZ and finally found out three genes which had the highest correlation: CXCR4, CXCL12 and Secreted Phosphoprotein 1 (SPP1). These three molecules are closely related to the tumor immune response. For example, Yu et al. (49) also demonstrated that CXCL12/CXCR4 is involved in the regulation of CRC proliferation. CXCL12/CXCR4 can sponge miR-133-a-3p and liberate its inhibitory effect on RhoA pathways, thus inducing the cytoskeletal remodeling and immunocytes recruitment, and promoting the progression of inflammatory CRC. Moreover, Zhang et al. (50) reported that SPP1 overexpression can positively regulate the expression of PD-L1, thereby promoting the macrophage polarization and immune escape in lung cancer. Interestingly, targeting the CXCR4, CXCL12, and SPP1 for CRC chemotherapy have been reported. For example, Jung et al. (51) demonstrated that bevacizumab and ramucirumab combined with the inhibitors AMD3100 of CXCR4 can improve the blockade in CRC angiogenesis. Cutler et al. (52) found that the chemotherapy drug 5-fluorouracil might inhibit tumor migration by selecting for CXCR4-negative cells in CRC. Moreover, SPP1 has been demonstrated to be significantly overexpressed in afatinib-resistant lung cancer cells, and that the knockdown of *SPP1* increases the chemotherapy sensitivity of lung cancer cells. These findings thus indicate that SPP1 can serve as a potential target for overcoming afatinib resistance (53). Consequently, we firmly believe that further studies on the correlation between TAZ and these co-expressed genes has the potential to provide new immunotherapy targets for CRC.

Interestingly, our KEGG analysis results revealed the correlation between TAZ expression and TGF-β signaling pathway, which has been reported to regulate extracellular matrix production, tissue fibrosis, and inflammation (54). Furthermore, the TGF-β signaling pathway is associated with the tumorigenesis, including EMT, immune escape and tumor angiogenesis (55). Li et al. (56) revealed that TAZ was involved in TGF-β1-induced EMT in oral cancer cells. Pseftogas et al. (57) explored the regulatory effect of CYLD in the hyperplastic alterations in the mammary epithelium of breast cancer, which is associated with the increased nuclear expression of TAZ and phosphorylation of Smad2/3, a key factor in TGF-β pathway, further supporting the involvement of TAZ in TGF-β-facilitated tumorigenesis. In view of the strong influence of TGF-β pathway on the formation of TME and tumor immune infiltration (58), the crosstalk between TAZ and TGF-β pathway may become a fresh entry point for targeted drug research. The correlation with “cytokine-cytokine receptor interaction” also indicates the potential mechanism of TAZ on the regulation of tumor immune landscape. Many cytokines such as interleukin play important roles in tumor immune process. IL-17 was reported to inhibit T cell attracting chemokines CXCL9 and CXCL10, thereby inhibiting the recruitment of CD8^+^ T cells and Treg cells in CRC, which helped the immune escape of tumor cells (59). Matsushita et al. (60) found that activated TAZ most significantly enhanced the transcription of genes related to “cytokine-cytokine receptor interaction” in malignant mesothelioma (MM). Their studies revealed that IL-1β induced by TAZ was a key factor for the development of malignant phenotype of mesothelioma cells.

Tumor immunotherapy has been widely investigated and employed in clinical fields, as it holds great promise as a neoadjuvant therapy besides surgery and radiotherapy (61–63). At present, many inhibitors have been identified to play emerging roles in tumor suppression by regulating TME or assisting immuno-surveillance by immune cells (64). Hippo pathway has is involved in the regulation of immune processes associated with tumorigenesis. Geng et al. (65) have shown that TAZ is a key co-activator of the Th17 transcription factor Rorγt, which is responsible for the differentiation of precursor CD4^+^ T cells into inflammatory Th17 cells. In contrast, TAZ has also been reported to suppress the acetylation of Foxp3 and inhibit the generation of Treg cells, suggesting that TAZ plays an important role in the lineage balance of Th17/Treg cells. *SKIL* is an important oncogene that regulates the induction of TGF-β pathway (66). Furthermore, Ma et al. (67) have reported that SKIL facilitates the immune escape of non-small cell lung cancer (NSCLC) cells by enhancing the expression of TAZ and activating the STRING pathway to induce the autophagy of malignant cells. In our study, we have determined that TAZ expression has significant correlation with the infiltration of CD4^+^ T, CD8^+^ T, and B cells in CRC using the TIMER database. Remarkably, our results demonstrate statistically significant correlation of TAZ expression with the expression CD8^+^ T cells, B cells, other T cells, macrophages, neutrophils, and monocyte gene markers. However, the specific mechanism illustrating the involvement of TAZ in regulating the tumor-associated immunological processes lacks evidence, and the effects of prospective application of TAZ-related inhibitors on CRC immunotherapy remain to be further explored.

In summary, our study provides comprehensive evidence that overexpression of TAZ in CRC tissues was highly correlated with the carcinogenesis and poor prognosis of CRC. Moreover, our co-expressed gene network and functional analysis indicated a notable correlation between TAZ expression and tumor metabolism as well as inflammation. Likewise, the expression of TAZ had a significant correlation with the immune infiltrating levels of CD4^+^ T, CD8^+^ T, and B cells, as well as the expression of immune cell specific gene markers in CRC tissues. Overall, our findings suggest that TAZ will become a novel biomarker in the diagnosis and treatment of CRC.

## Data Availability Statement

The original contributions presented in this study are included in the article/Supplementary Material, further inquiries can be directed to the corresponding author.

## Ethics Statement

The studies involving human participants were reviewed and approved by the Ethics Committee of the Xiangya Hospital, Central South University. The patients/participants provided their written informed consent to participate in this study.

## Author Contributions

YW and HN conducted experimental operations, sample processing, data analysis, and performed the experiments. CO conceived and designed the experiments. All authors participated in writing the manuscript, read, and approved the final manuscript.

## Conflict of Interest

The authors declare that the research was conducted in the absence of any commercial or financial relationships that could be construed as a potential conflict of interest.

## Publisher’s Note

All claims expressed in this article are solely those of the authors and do not necessarily represent those of their affiliated organizations, or those of the publisher, the editors and the reviewers. Any product that may be evaluated in this article, or claim that may be made by its manufacturer, is not guaranteed or endorsed by the publisher.

## Footnotes

1
http://cistrome.shinyapps.io/timer/

2
http://ualcan.path.uab.edu/index.html

3
http://cbioportal.org

4
https://cytoscape.org

5
http://metascape.org

## References

[1] Taha Z Janse van Rensburg HJ Yang X. The hippo pathway: immunity and cancer. *Cancers.* (2018) 10:94. 10.3390/cancers10040094 29597279 PMC5923349

[2] Ou C Sun Z Li X Li X Ren W Qin Z MiR-590-5p, a density-sensitive microRNA, inhibits tumorigenesis by targeting YAP1 in colorectal cancer. *Cancer Lett.* (2017) 399:53–63. 10.1016/j.canlet.2017.04.011 28433598

[3] Harvey KF Zhang X Thomas DM. The hippo pathway and human cancer. *Nat Rev Cancer.* (2013) 13:246–57. 10.1038/nrc3458 23467301

[4] Ou C Sun Z He X Li X Fan S Zheng X Targeting YAP1/LINC00152/FSCN1 signaling axis prevents the progression of colorectal cancer. *Adv Sci.* (2019) 7:1901380. 10.1002/advs.201901380 32042551 PMC7001651

[5] Lai CJ Lin CY Liao WY Hour TC Wang HD Chuu CP. CD44 promotes migration and invasion of docetaxel-resistant prostate cancer cells likely via induction of hippo-yap signaling. *Cells.* (2019) 8:295. 10.3390/cells8040295 30935014 PMC6523775

[6] Muñoz-Galván S Felipe-Abrio B Verdugo-Sivianes EM Perez M Jiménez-García MP Suarez-Martinez E Downregulation of MYPT1 increases tumor resistance in ovarian cancer by targeting the hippo pathway and increasing the stemness. *Mol Cancer.* (2020) 19:7. 10.1186/s12943-020-1130-z 31926547 PMC6954568

[7] Shen J Cao B Wang Y Ma C Zeng Z Liu L Hippo component YAP promotes focal adhesion and tumour aggressiveness via transcriptionally activating THBS1/FAK signalling in breast cancer. *J Exp Clin Cancer Res.* (2018) 37:175. 10.1186/s13046-018-0850-z 30055645 PMC6064138

[8] Zheng YW Li ZH Lei L Liu CC Wang Z Fei LR FAM83A promotes lung cancer progression by regulating the wnt and hippo signaling pathways and indicates poor prognosis. *Front Oncol.* (2020) 10:180. 10.3389/fonc.2020.00180 32195172 PMC7066079

[9] Piccolo S Dupont S Cordenonsi M. The biology of YAP/TAZ: hippo signaling and beyond. *Physiol Rev.* (2014) 94:1287–312. 10.1152/physrev.00005.2014 25287865

[10] Maugeri-Saccà M Barba M Pizzuti L Vici P Di Lauro L Dattilo R The Hippo transducers TAZ and YAP in breast cancer: oncogenic activities and clinical implications. *Expert Rev Mol Med.* (2015) 17:e14. 10.1017/erm.2015.12 26136233

[11] Bhandari A Guan Y Xia E Huang Q Chen Y. VASN promotes YAP/TAZ and EMT pathway in thyroid carcinogenesis in vitro. *Am J Transl Res.* (2019) 11:3589–99. 31312369 PMC6614637

[12] Edwards DN Ngwa VM Wang S Shiuan E Brantley-Sieders DM Kim LC The receptor tyrosine kinase EphA2 promotes glutamine metabolism in tumors by activating the transcriptional coactivators YAP and TAZ. *Sci Signal.* (2017) 10:eaan4667. 10.1126/scisignal.aan4667 29208682 PMC5819349

[13] Yuan W Xu W Li Y Jiang W Li Y Huang Q TAZ sensitizes EGFR wild-type non-small-cell lung cancer to gefitinib by promoting amphiregulin transcription. *Cell Death Dis.* (2019) 10:283. 10.1038/s41419-019-1519-z 30911072 PMC6433914

[14] Van Haele M Moya IM Karaman R Rens G Snoeck J Govaere O YAP and TAZ heterogeneity in primary liver cancer: an analysis of its prognostic and diagnostic role. *Int J Mol Sci.* (2019) 20:638. 10.3390/ijms20030638 30717258 PMC6386931

[15] Chen X Wang AL Liu YY Zhao CX Zhou X Liu HL MiR-429 involves in the pathogenesis of colorectal cancer via directly targeting LATS2. *Oxid Med Cell Longev.* (2020) 2020:5316276. 10.1155/2020/5316276 33414893 PMC7769676

[16] Wang L Shi S Guo Z Zhang X Han S Yang A Overexpression of YAP and TAZ is an independent predictor of prognosis in colorectal cancer and related to the proliferation and metastasis of colon cancer cells. *PLoS One.* (2013) 8:e65539. 10.1371/journal.pone.0065539 23762387 PMC3677905

[17] Yuen HF McCrudden CM Huang YH Tham JM Zhang X Zeng Q TAZ expression as a prognostic indicator in colorectal cancer. *PLoS One.* (2013) 8:e54211. 10.1371/journal.pone.0054211 23372686 PMC3553150

[18] Yoo G Park D Kim Y Chung C. New insights into the clinical implications of yes-associated protein in lung cancer: roles in drug resistance, tumor immunity, autophagy, and organoid development. *Cancers.* (2021) 13:3069. 10.3390/cancers13123069 34202980 PMC8234989

[19] Murakami S Shahbazian D Surana R Zhang W Chen H Graham GT Yes-associated protein mediates immune reprogramming in pancreatic ductal adenocarcinoma. *Oncogene.* (2017) 36:1232–44. 10.1038/onc.2016.288 27546622 PMC5322249

[20] Pan Z Tian Y Cao C Niu G. The emerging role of YAP/TAZ in tumor immunity. *Mol Cancer Res.* (2019) 17:1777–86. 10.1158/1541-7786.MCR-19-0375 31308148

[21] Janse van Rensburg HJ Azad T Ling M Hao Y Snetsinger B Khanal P The hippo pathway component TAZ promotes immune evasion in human cancer through PD-L1. *Cancer Res.* (2018) 78:1457–70. 10.1158/0008-5472.CAN-17-31399 29339539

[22] Tong K Zhu W Fu H Cao F Wang S Zhou W Frequent KRAS mutations in oncocytic papillary renal neoplasm with inverted nuclei. *Histopathology.* (2020) 76:1070–83. 10.1111/his.14084 31997427

[23] Zheng H Zhan Y Zhang Y Liu S Lu J Yang Y Elevated expression of G3BP1 associates with YB1 and p-AKT and predicts poor prognosis in nonsmall cell lung cancer patients after surgical resection. *Cancer Med.* (2019) 8:6894–903. 10.1002/cam4.2579 31560169 PMC6853815

[24] Hong Y Downey T Eu KW Koh PK Cheah PY. A ‘metastasis-prone’ signature for early-stage mismatch-repair proficient sporadic colorectal cancer patients and its implications for possible therapeutics. *Clin Exp Metastasis.* (2010) 27:83–90. 10.1007/s10585-010-9305-4 20143136

[25] Okazaki S Ishikawa T Iida S Ishiguro M Kobayashi H Higuchi T Clinical significance of UNC5B expression in colorectal cancer. *Int J Oncol.* (2012) 40:209–16. 10.3892/ijo.2011.1201 21922135

[26] Matsuyama T Ishikawa T Mogushi K Yoshida T Iida S Uetake H MUC12 mRNA expression is an independent marker of prognosis in stage II and stage III colorectal cancer. *Int J Cancer.* (2010) 127:2292–9. 10.1002/ijc.25256 20162577

[27] Marisa L de Reyniès A Duval A Selves J Gaub MP Vescovo L Gene expression classification of colon cancer into molecular subtypes: characterization, validation, and prognostic value. *PLoS Med.* (2013) 10:e1001453. 10.1371/journal.pmed.1001453 23700391 PMC3660251

[28] Li T Fan J Wang B Traugh N Chen Q Liu JS TIMER: a web server for comprehensive analysis of tumor-infiltrating immune cells. *Cancer Res.* (2017) 77:e108–10. 10.1158/0008-5472.CAN-17-0307 29092952 PMC6042652

[29] Danaher P Warren S Dennis L D’Amico L White A Disis ML Gene expression markers of tumor infiltrating leukocytes. *J Immunother Cancer.* (2017) 5:18. 10.1186/s40425-017-0215-8 28239471 PMC5319024

[30] Chandrashekar DS Bashel B Balasubramanya SAH Creighton CJ Ponce-Rodriguez I Chakravarthi BVSK UALCAN: a portal for facilitating tumor subgroup gene expression and survival analyses. *Neoplasia.* (2017) 19:649–58. 10.1016/j.neo.2017.05.002 28732212 PMC5516091

[31] Cerami E Gao J Dogrusoz U Gross BE Sumer SO Aksoy BA The cBio cancer genomics portal: an open platform for exploring multidimensional cancer genomics data. *Cancer Discov.* (2012) 2:401–4. 10.1158/2159-8290.CD-12-0095 22588877 PMC3956037

[32] Su G Morris JH Demchak B Bader GD. Biological network exploration with Cytoscape 3. *Curr Protoc Bioinformatics.* (2014) 47:8.13.1–24. 10.1002/0471250953.bi0813s47 25199793 PMC4174321

[33] Zhou Y Zhou B Pache L Chang M Khodabakhshi AH Tanaseichuk O Metascape provides a biologist-oriented resource for the analysis of systems-level datasets. *Nat Commun.* (2019) 10:1523. 10.1038/s41467-019-09234-6 30944313 PMC6447622

[34] Tang Z Li C Kang B Gao G Li C Zhang Z. Gepia: a web server for cancer and normal gene expression profiling and interactive analyses. *Nucleic Acids Res.* (2017) 45:W98–102. 10.1093/nar/gkx247 28407145 PMC5570223

[35] Mouillet-Richard S Laurent-Puig P. YAP/TAZ signalling in colorectal cancer: lessons from consensus molecular subtypes. *Cancers.* (2020) 12:3160. 10.3390/cancers12113160 33126419 PMC7692643

[36] Zhang SD McCrudden CM Yuen HF Leung KL Hong WJ Kwok HF. Association between the expression levels of TAZ, AXL and CTGF and clinicopathological parameters in patients with colon cancer. *Oncol Lett.* (2016) 11:1223–9. 10.3892/ol.2015.3999 26893723 PMC4734239

[37] Alspach E Lussier DM Miceli AP Kizhvatov I DuPage M Luoma AM MHC-II neoantigens shape tumour immunity and response to immunotherapy. *Nature.* (2019) 574:696–701. 10.1038/s41586-019-1671-8 31645760 PMC6858572

[38] Honkala AT Tailor D Malhotra SV. Guanylate-binding protein 1: an emerging target in inflammation and cancer. *Front Immunol.* (2020) 24:3139. 10.3389/fimmu.2019.03139 32117203 PMC7025589

[39] Engelhard VH Rodriguez AB Mauldin IS Woods AN Peske JD Slingluff CL Jr Immune cell infiltration and tertiary lymphoid structures as determinants of antitumor immunity. *J Immunol.* (2018) 200:432–42. 10.4049/jimmunol.1701269 29311385 PMC5777336

[40] Liao Z Nie H Wang Y Luo J Zhou J Ou C. The emerging landscape of long non-coding RNAs in colorectal cancer metastasis. *Front Oncol.* (2021) 11:641343. 10.3389/fonc.2021.641343 33718238 PMC7947863

[41] Wang Y Nie H He X Liao Z Zhou Y Zhou J The emerging role of super enhancer-derived noncoding RNAs in human cancer. *Theranostics.* (2020) 10:11049–62. 10.7150/thno.49168 33042269 PMC7532672

[42] Wang Y He X Nie H Zhou J Cao P Ou C. Application of artificial intelligence to the diagnosis and therapy of colorectal cancer. *Am J Cancer Res.* (2020) 10:3575–98. 33294256 PMC7716173

[43] Mazzotta E Villalobos-Hernandez EC Fiorda-Diaz J Harzman A Christofi FL. Postoperative ileus and postoperative gastrointestinal tract dysfunction: pathogenic mechanisms and novel treatment strategies beyond colorectal enhanced recovery after surgery protocols. *Front Pharmacol.* (2020) 11:583422. 10.3389/fphar.2020.583422 33390950 PMC7774512

[44] Nie H Wang Y Liao Z Zhou J Ou C. The function and mechanism of circular RNAs in gastrointestinal tumours. *Cell Prolif.* (2020) 53:e12815. 10.1111/cpr.12815 32515024 PMC7377939

[45] Wang Y Xu X Maglic D Dill MT Mojumdar K Ng PK Comprehensive molecular characterization of the hippo signaling pathway in cancer. *Cell Rep.* (2018) 25:1304–17.e5. 10.1016/j.celrep.2018.10.001 30380420 PMC6326181

[46] Ros J Baraibar I Martini G Salvà F Saoudi N Cuadra-Urteaga JL The evolving role of consensus molecular subtypes: a step beyond inpatient selection for treatment of colorectal cancer. *Curr Treat Options Oncol.* (2021) 22:113. 10.1007/s11864-021-00913-5 34741675

[47] Wang H Liu J Li J Zang D Wang X Chen Y Identification of gene modules and hub genes in colon adenocarcinoma associated with pathological stage based on WGCNA analysis. *Cancer Genet.* (2020) 242:1–7. 10.1016/j.cancergen.2020.01.052 32036224

[48] Wang Y Chen L Wang G Cheng S Qian K Liu X Fifteen hub genes associated with progression and prognosis of clear cell renal cell carcinoma identified by coexpression analysis. *J Cell Physiol.* (2019) 234:10225–37. 10.1002/jcp.27692 30417363

[49] Yu X Wang D Wang X Sun S Zhang Y Wang S CXCL12/CXCR4 promotes inflammation-driven colorectal cancer progression through activation of RhoA signaling by sponging miR-133a-3p. *J Exp Clin Cancer Res.* (2019) 38:32. 10.1186/s13046-018-1014-x 30678736 PMC6346552

[50] Zhang Y Du W Chen Z Xiang C. Upregulation of PD-L1 by SPP1 mediates macrophage polarization and facilitates immune escape in lung adenocarcinoma. *Exp Cell Res.* (2017) 359:449–57. 10.1016/j.yexcr.2017.08.028 28830685

[51] Jung K Heishi T Incio J Huang Y Beech EY Pinter M Targeting CXCR4-dependent immunosuppressive Ly6Clow monocytes improves antiangiogenic therapy in colorectal cancer. *Proc Natl Acad Sci U S A.* (2017) 114:10455–60. 10.1073/pnas.1710754114 28900008 PMC5625928

[52] Cutler MJ Lowthers EL Richard CL Hajducek DM Spagnuolo PA Blay J. Chemotherapeutic agents attenuate CXCL12-mediated migration of colon cancer cells by selecting for CXCR4-negative cells and increasing peptidase CD26. *BMC Cancer.* (2015) 15:882. 10.1186/s12885-015-1702-2 26552750 PMC4640216

[53] Wang X Zhang F Yang X Xue M Li X Gao Y Secreted Phosphoprotein 1 (SPP1) contributes to second-generation EGFR tyrosine kinase inhibitor resistance in non-small cell lung cancer. *Oncol Res.* (2019) 27:871–7. 10.3727/096504018X15426271404407 30832751 PMC7848392

[54] Hata A Chen YG. TGF-β signaling from receptors to Smads. *Cold Spring Harb Perspect Biol.* (2016) 8:a022061. 10.1101/cshperspect.a022061 27449815 PMC5008074

[55] Hao Y Baker D Ten Dijke P. TGF-β-mediated epithelial-mesenchymal transition and cancer metastasis. *Int J Mol Sci.* (2019) 20:2767. 10.3390/ijms20112767 31195692 PMC6600375

[56] Li Z Wang Y Zhu Y Yuan C Wang D Zhang W The Hippo transducer TAZ promotes epithelial to mesenchymal transition and cancer stem cell maintenance in oral cancer. *Mol Oncol.* (2015) 9:1091–105. 10.1016/j.molonc.2015.01.007 25704916 PMC5528756

[57] Pseftogas A Xanthopoulos K Poutahidis T Ainali C Dafou D Panteris E The tumor suppressor CYLD inhibits mammary epithelial to mesenchymal transition by the coordinated inhibition of YAP/TAZ and TGFβ signaling. *Cancers.* (2020) 12:2047. 10.3390/cancers12082047 32722292 PMC7466024

[58] Zhao H Wei J Sun J. Roles of TGF-β signaling pathway in tumor microenvirionment and cancer therapy. *Int Immunopharmacol.* (2020) 89:107101. 10.1016/j.intimp.2020.107101 33099067

[59] Chen J Ye X Pitmon E Lu M Wan J Jellison ER IL-17 inhibits CXCL9/10-mediated recruitment of CD8+ cytotoxic T cells and regulatory T cells to colorectal tumors. *J Immunother Cancer.* (2019) 7:324. 10.1186/s40425-019-0757-z 31775909 PMC6880503

[60] Matsushita A Sato T Mukai S Fujishita T Mishiro-Sato E Okuda M TAZ activation by hippo pathway dysregulation induces cytokine gene expression and promotes mesothelial cell transformation. *Oncogene.* (2019) 38:1966–78. 10.1038/s41388-018-0417-7 30401981

[61] Peng L Wang D Han Y Huang T He X Wang J Emerging role of cancer-associated fibroblasts-derived exosomes in tumorigenesis. *Front Immunol.* (2022) 12:795372. 10.3389/fimmu.2021.795372 35058933 PMC8764452

[62] Zhou H He X He Y Ou C Cao P. Exosomal circRNAs: emerging players in tumor metastasis. *Front Cell Dev Biol.* (2021) 9:786224. 10.3389/fcell.2021.786224 34957113 PMC8692866

[63] Han Y Wang D Peng L Huang T He X Wang J Single-cell sequencing: a promising approach for uncovering the mechanisms of tumor metastasis. *J Hematol Oncol.* (2022) 15:59. 10.1186/s13045-022-01280-w 35549970 PMC9096771

[64] Li X Wenes M Romero P Huang SC Fendt SM Ho PC. Navigating metabolic pathways to enhance antitumour immunity and immunotherapy. *Nat Rev Clin Oncol.* (2019) 16:425–41. 10.1038/s41571-019-0203-7 30914826

[65] Geng J Yu S Zhao H Sun X Li X Wang P The transcriptional coactivator TAZ regulates reciprocal differentiation of T_*H*_17 cells and T_*reg*_ cells. *Nat Immunol.* (2017) 18:800–12. 10.1038/ni.3748 28504697

[66] Tecalco-Cruz AC Sosa-Garrocho M Vázquez-Victorio G Ortiz-García L Domínguez-Hüttinger E Macías-Silva M. Transforming growth factor-β/SMAD target gene SKIL is negatively regulated by the transcriptional cofactor complex SNON-SMAD4. *J Biol Chem.* (2012) 287:26764–76. 10.1074/jbc.M112.386599 22674574 PMC3411014

[67] Ma F Ding MG Lei YY Luo LH Jiang S Feng YH SKIL facilitates tumorigenesis and immune escape of NSCLC via upregulating TAZ/autophagy axis. *Cell Death Dis.* (2020) 11:1028. 10.1038/s41419-020-03200-7 33268765 PMC7710697

