# Supplementary material for: The Hippo Pathway Effector Transcriptional Co-activator With PDZ-Binding Motif Correlates With Clinical Prognosis and Immune Infiltration in Colorectal Cancer
Source: Front Med (Lausanne). 2022 Jul 5;9:888093. doi: 10.3389/fmed.2022.888093 (PMC9295930; doi:10.3389/fmed.2022.888093)
Supplement: Supplementary file 2 [file Table_2.DOCX]

**Supplementary Table 2** Association between the expression of TAZ and CRC patients’ clinicopathological parameters

| **Characteristics** | **N** | **TAZ** | | **High(%)** | **χ2** | ***P*** |
| --- | --- | --- | --- | --- | --- | --- |
|  |  | Low | High |  |  |  |
| BRAF status |  |  |  |  | - | 0.630 |
| Wild-type | 62 | 23 | 39 | 62.90% |  |  |
| Mutation | 4 | 2 | 2 | 50.00% |  |  |
| KRAS |  |  |  |  | 0.326 | 0.569 |
| Wild-type | 26 | 10 | 16 | 61.53% |  |  |
| Mutation | 38 | 12 | 26 | 68.42% |  |  |
| MSI |  |  |  |  | 0.430 | 0.512 |
| MSS | 75 | 33 | 42 | 56.00% |  |  |
| MSI-H | 17 | 6 | 11 | 64.71% |  |  |
