# Supplementary material for: The Hippo Pathway Effector Transcriptional Co-activator With PDZ-Binding Motif Correlates With Clinical Prognosis and Immune Infiltration in Colorectal Cancer
Source: Front Med (Lausanne). 2022 Jul 5;9:888093. doi: 10.3389/fmed.2022.888093 (PMC9295930; doi:10.3389/fmed.2022.888093)
Supplement: Supplementary file 4 [file Table_4.DOCX]

**Supplementary Table 4** The association between the expression of TAZ and the markers of immune cells

|  |  | TAZ | | | |  |  | TAZ | | | |
| --- | --- | --- | --- | --- | --- | --- | --- | --- | --- | --- | --- |
|  |  | COAD | | READ | |  |  | COAD | | READ | |
|  |  | Cor | *p* | Cor | *p* |  |  | Cor | *p* | Cor | *p* |
| CD8+ Tcell | CD8A | -0.111 | *** | -0.054 | 0.501 | T cell | CD3D | -0.153 | *** | 0.005 | 0.957 |
|  | CD8B | -0.100 | * | -0.010 | 0.899 |  | CD3E | -0.140 | ** | -0.049 | 0.530 |
|  | GZMA | -0.250 | *** | -0.014 | 0.868 |  | CD2 | -0.120 | *** | -0.026 | 0.071 |
| B cell | CD19 | 0.135 | 0.756 | -0.049 | 0.528 | TAM | CCL2 | -0.205 | *** | -0.087 | 0.264 |
|  | CD79A | -0.025 | 0.597 | -0.154 | * |  | CD68 | -0.123 | ** | 0.040 | 0.605 |
|  | MS4A1 | 0.020 | 0.124 | -0.131 | * |  | IL10 | -0.193 | *** | -0.015 | 0.850 |
| M1 | IRF5 | 0.230 | *** | 0.320 | *** | M2 | MS4A4A | -0.259 | *** | -0.056 | 0.477 |
|  | PTGS2 | -0.220 | *** | -0.120 | 0.123 |  | CD163 | -0.163 | *** | 0.112 | 0.189 |
|  | NOS2 | -0.083 | 0.074 | 0.036 | 0.641 |  | VSIG4 | -0.195 | *** | 0.101 | 0.236 |
| Neutrophils | ITGAM | -0.116 | ** | 0.115 | 0.176 | Monocyte | CD86 | -0.260 | *** | -0.102 | 0.189 |
|  | CCR7 | -0.045 | 0.341 | -0.063 | 0.416 |  | C3AR1 | -0.195 | *** | 0.128 | 0.139 |
|  | SIGLEC5 | -0.158 | *** | 0.175 | ** |  | CSF1R | -0.111 | *** | 0.163 | 0.055 |
| DC | HLA-DQB1 | -0.185 | *** | 0.012 | 0.879 | NK cell | KIR2DL1 | -0.150 | ** | -0.092 | 0.240 |
|  | HLA-DPB1 | -0.203 | *** | -0.051 | 0.515 |  | KIR2DL3 | -0.146 | ** | -0.035 | 0.653 |
|  | HLA-DRA | -0.266 | *** | -0.115 | 0.141 |  | KIR2DL4 | -0.223 | *** | 0.026 | 0.735 |
|  | HLA-DPA1 | -0.228 | *** | -0.130 | 0.096 |  | KIR3DL1 | -0.168 | *** | -0.104 | 0.181 |
|  | ITGAX | -0.082 | 0.078 | 0.089 | 0.256 |  | KIR3DL2 | -0.197 | *** | -0.020 | 0.794 |
|  | CD1C | -0.132 | ** | -0.108 | 0.166 |  | KIR3DL3 | -0.113 | * | -0.011 | 0.892 |
|  | NRP1 | -0.151 | ** | -0.167 | * |  | KIR2DS4 | -0.163 | *** | -0.038 | 0.625 |
| Th1 | TBX21 | -0.121 | * | -0.019 | 0.811 | Th2 | STAT6 | 0.173 | *** | 0.105 | 0.179 |
|  | STAT1 | -0.111 | * | -0.141 | 0.071 |  | GATA3 | -0.040 | 0.390 | -0.115 | 0.140 |
|  | STAT4 | -0.174 | *** | -0.084 | 0.279 |  | STAT5A | 0.041 | 0.381 | -0.003 | 0.966 |
|  | IFNG | -0.123 | ** | -0.154 | * |  | IL13 | -0.081 | 0.084 | 0.011 | 0.885 |
| Tfh | BCL6 | -0.076 | 0.106 | -0.080 | 0.302 | Th17 | STAT3 | -0.126 | ** | -0.079 | 0.312 |
|  | IL21 | -0.076 | 0.105 | -0.247 | ** |  | IL17A | 0.094 | * | 0.011 | 0.885 |
| Treg | FOXP3 | -0.048 | 0.305 | 0.035 | 0.651 | T exhaustion-cell | PDCD1 | -0.099 | * | -0.076 | 0.331 |
|  | STAT5B | 0.309 | *** | 0.062 | 0.428 |  | CTLA4 | -0.143 | ** | -0.025 | 0.752 |
|  | CCR8 | -0.132 | ** | -0.121 | 0.121 |  | HAVCR2 | -0.253 | *** | -0.027 | 0.729 |
|  | TGFB1 | -0.139 | ** | 0.045 | 0.564 |  | LAG3 | -0.108 | * | -0.021 | 0.792 |
|  |  |  |  |  |  |  |  |  |  |  |  |
