# Supplementary figures and images for: The Hippo Pathway Effector Transcriptional Co-activator With PDZ-Binding Motif Correlates With Clinical Prognosis and Immune Infiltration in Colorectal Cancer
Source: Front Med (Lausanne). 2022 Jul 5;9:888093. doi: 10.3389/fmed.2022.888093 (PMC9295930; doi:10.3389/fmed.2022.888093)

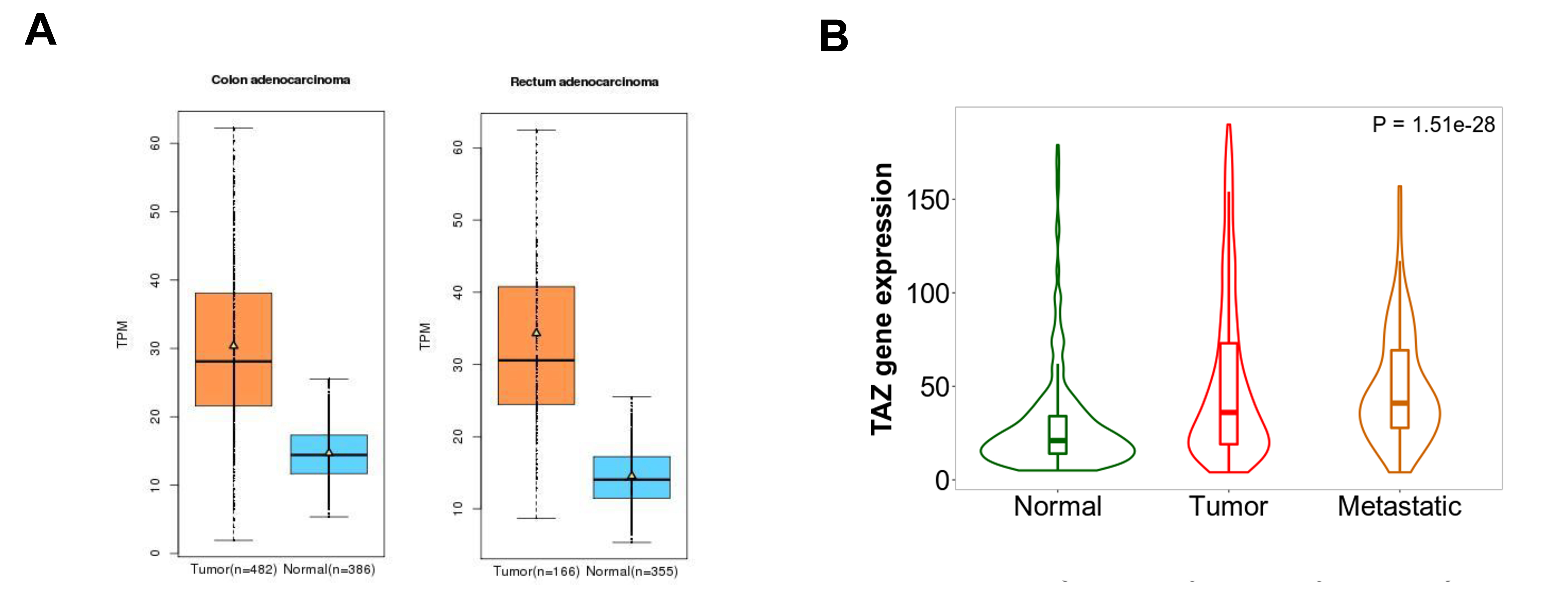

Supplement: Supplementary file 5 [file Image_1.JPEG]

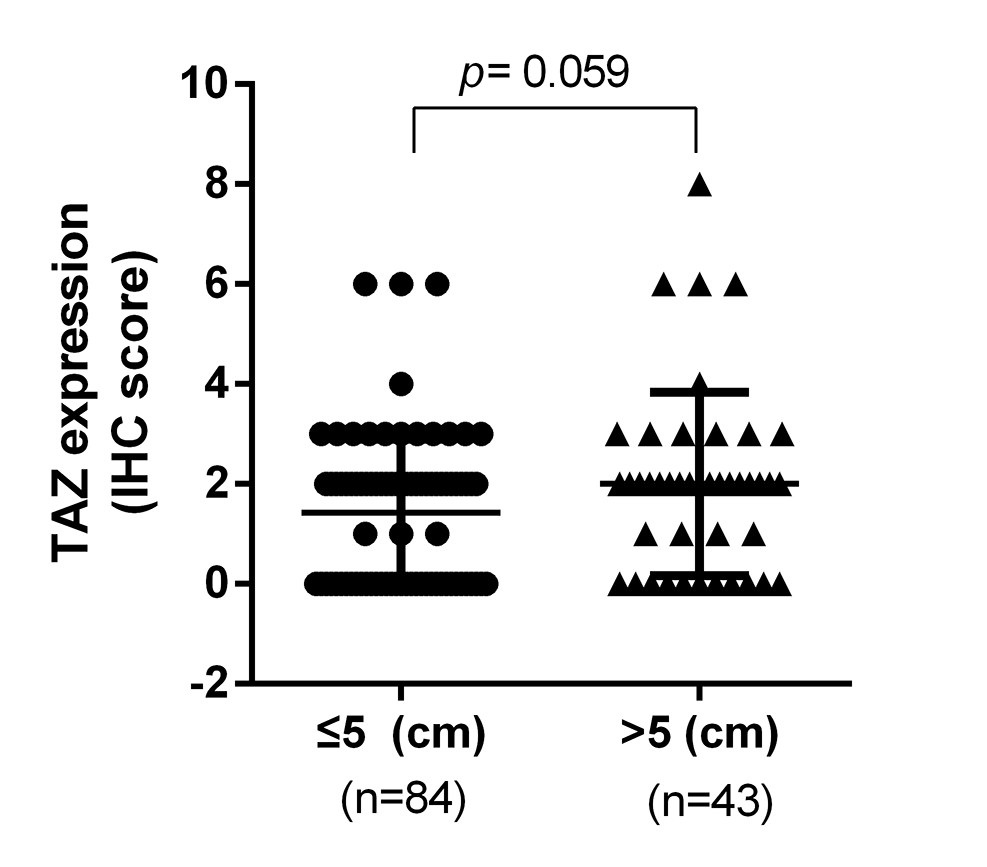

Supplement: Supplementary file 6 [file Image_2.TIF]
